# A Mixed-Methods Investigation of the Effectiveness and Perceptions of Learning English Collocations Using the Keyword Method and the Rote Learning Method

**DOI:** 10.3390/bs13070591

**Published:** 2023-07-14

**Authors:** Xiaofang Zhang, Barry Lee Reynolds

**Affiliations:** 1 Faculty of Education, University of Macau, Room 1014, E33, Av. da Universidade, Taipa, Macau SAR, China; yc17111@umac.mo; 2 Centre for Cognitive and Brain Sciences, University of Macau, Av. da Universidade, Taipa, Macau SAR, China

**Keywords:** vocabulary learning strategies (VLSs), keyword method, rote learning, collocations, mixed-methods

## Abstract

This study investigated the effectiveness, as well as EFL learners’ perceptions, of the keyword method (KWM) in comparison to the rote learning method (RLM) for the learning of English collocations. A controlled laboratory-like setting was adopted for randomly assigning participants to the KWM group (*n* = 15) or the RLM group (*n* = 15). After receiving training on the use of the respective strategy, the two participant groups applied the respective strategy to the learning of collocations. Collocations were assessed at three different time periods, and additional data regarding perceptions of the two strategies were elicited through one-on-one post hoc interviews. The quantitative data revealed that the KWM was superior to the RLM in terms of the long-term retention of productive collocation knowledge; knowledge of adjective–noun collocations was retained better than verb–noun collocations. The qualitative data revealed that participants deemed that the KWM was unfamiliar but effective. Additionally, participants claimed that the RLM was facile but may result in a high rate of forgetting. The pedagogical implications are that foreign language teachers should encourage language learners to use the KWM for learning English collocations. Although the KWM has been recommend by many researchers, it is still rarely advocated for by foreign language instructors. Therefore, it is important that both EFL learners and teachers should be aware of the KWM’s long-term retention effects on the learning of English collocations and apply this vocabulary learning strategy (VLS) in their actual learning and teaching context.

## 1. Introduction

The value of collocation learning and teaching has gradually become an acknowledged facet of not only the second language (L2) vocabulary research field but also the more encompassing field of second language acquisition (SLA) [1,2,3,4]. According to Henriksen [5], collocations are “frequently recurring two-to-three word syntagmatic units which can include both lexical and grammatical words” (p. 30), for example, verb–noun (*risk neck*) and adjective–noun (*broad daylight*) collocations. Collocations are commonly characterized based on formal and functional features [6]. With respect to their formal features, two elements are integrated: the node and the collocate. In relation to their functional features, collocations are structured combinations of words. Two types of collocations are of interest to this study, namely, verb–noun and adjective–noun collocations, as they are the most researched types of collocations in previous studies because of their ubiquitous appearance in language [5,7] and, thus, can allow for comparisons to previously published research. Furthermore, studies have shown that verb–noun collocations “tend to be particularly problematic for language learners” [2]. Also, adjective–noun collocations deserve more attention, as they have traditionally been neglected in previous collocation studies [6]. Although numerous studies [1,8,9,10] have investigated the effects of L2 instructional methods on the learning of English collocations, few useful EFL collocation learning strategies or teaching guidelines are available [11]. Frequently appearing in relevant discussions is that EFL learners have difficulties in accurately producing collocations [10,12,13], and the inaccurate production of collocations may result in non-native and nonidiomatic language production [14] that affects comprehension [15]. Although researchers such as Lewis [16] suggested some general collocation classroom techniques aimed at raising teachers’ and students’ awareness of the necessity of teaching and learning collocations, an unimpressive number of empirical studies have been conducted to determine whether these suggestions are effective. Even more surprising is that few researchers have suggested the use of mnemonics as a useful method for English collocation learning [17,18,19], even though previous studies have found that mnemonics facilitate learners’ vocabulary learning [20,21].

The keyword method (KWM) is one of many mnemonic vocabulary learning strategies (VLSs) used for deliberate vocabulary learning. The KWM is an image mnemonic that combines two items together, so one item will be recalled when the other item is thought about. Atkinson [22] explained that the KWM involves a two-stage process. Stage one is to associate an L2 foreign word with an L1 keyword that sounds like part of the L2 foreign word, while stage two entails the learner “form[ing] a mental image of the keyword ‘interacting’ with the” L1 definition [22] (p. 2). For example, the English word “beauty” sounds like the Mandarin Chinese word “bítì” (meaning “snot”), so we may employ the word “bítì” as the keyword that evokes the image: “[t]he beauty is blowing snot out of her nose”. Although a sizable number of studies [14,23,24,25,26,27,28,29,30,31] have investigated the effectiveness of the KWM for vocabulary learning, a precursory look at the published literature shows that it has been underrated by language learners and teachers and under researched by SLA scholars. As one of many mnemonics used by language learners to assist in the deliberate learning of vocabulary, the KWM may have the potential to aid in the long-term retention of English collocations. Furthermore, as most first-language (L1) Chinese speakers use rote learning to master L2 collocations [32], it would be interesting to know whether the commonly applied rote learning method (RLM) would be as effective as the use of the KWM for the learning of English collocations. In the SLA literature, the RLM can contribute to the “acquisition, storage or retrieval” [33] (p. 235) of new language knowledge and has been understood as one of the many VLSs that cover repetition, practice, and memorization [32]. Taking the English word “beauty”, again, as an example, leaners using the RLM would first look at the L2 word’s spelling, then look at the L1 definition, and finally read and then write down the target word and its L1 definition repeatedly. In fact, previous research has been conducted in which the effectiveness of the RLM for individual word learning has been compared to different mnemonic VLSs (e.g., the KWM [23,24], using flash cards [34], and context learning [35]). Unlike learning by rote, when applying the KWM, learners need to identify the keyword and create an interactive image between the keyword and the foreign word, so the RLM can be considered a more general language learning strategy, while the KWM can be considered a more specific vocabulary learning strategy (VLS) [27,36,37]. Therefore, it is important to compare these two strategies’ effectiveness for collocation learning to enrich research findings for the field of vocabulary studies.

While many facets of the KWM have been previously studied, there are several variables that have been overlooked. Firstly, most previous studies [21,28,31,38] focused on collecting quantitative data; however, qualitative research findings on the effectiveness of the KWM are scarce [39,40]. Secondly, most extant studies only assessed one type of vocabulary knowledge, even though research has shown that vocabulary acquisition is incremental and should be assessed using multiple methods [40]. Previous literature has identified that vocabulary learning is incremental, because the mastery of vocabulary is gradual, and learners need to be exposed to the target vocabulary multiple times [41,42]. Specifically, the learning of vocabulary knowledge is not all or nothing, so the measurements of two types of vocabulary knowledge is necessary. Thirdly, researchers underestimated the value of the KWM for L1 Mandarin Chinese speakers, claiming difficulties in selecting Mandarin Chinese keywords [14]; this fallacy deserves close scrutiny because less is known about learning English collocations using Chinese keywords [14]. Fourthly, previous studies [43,44] mostly examined the immediate recall of vocabulary knowledge but without further investigating the KWM’s effect on the long-term retention of vocabulary knowledge. Taking all these issues into consideration, a mixed-methods study design was adopted to address these research gaps.

### The Present Study

Thirty Mandarin-L1 EFL college students were randomly assigned to one of the two groups: RLM group (*n* = 15) and KWM group (*n* = 15). The learning of ten target collocations, including five verb–noun collocations and five adjective–noun collocations, was assessed at three different time periods (pre-test, immediate post-test, and delayed post-test) using two different measures of collocation knowledge: receptive recognition and productive recall. Additional data elicited from interviews with the two groups were gathered after the delayed post-test to gain a deeper understanding of participants’ perceptions of the two VLSs. The main purpose of this study was to investigate the effectiveness, as well as learners’ perceptions, of the KWM in comparison to the RLM for the learning of English collocations. More specifically, this study aimed to answer the following three research questions:

RQ1: Which VLS is more effective for learning English collocations: KWM or RLM?

RQ2: Which type of English collocations can be learned more effectively by using the KWM: verb–noun or adjective–noun collocations?

RQ3: How did learners perceive the KWM and the RLM for learning English collocations?

## 2. Literature Review

### 2.1. Importance of Vocabulary Knowledge for EFL Learners in General and Collocations in Particular

The understanding of the importance of collocations to L2 learning has been gradually enriched and amplified based on previous studies [44,45,46,47]. According to Henriksen [5], collocations are “frequently recurring two-to-three word syntagmatic units which can include both lexical and grammatical words” (p. 30), for example, verb–noun (e.g., risk neck) and adjective–noun (e.g., broad daylight) collocations. Collocations can be considered congruent if a target collocation’s concept can be expressed with a direct translation into the L1 [48]. Different from congruent collocations, incongruent collocations have no direct translation between the learner’s L1 and L2. Therefore, incongruent collocations are more difficult to learn than congruent collocations.

Collocations have been researched from various perspectives (e.g., the mastery of collocations for communicative needs [5]; L2 learners’ collocational competence [5]). This study limited the focus to learning collocations through the application of language learning strategies. Mastery of collocations is a key aspect for learners’ communicative competence [49] and the proper use of collocations is now widely accepted as a prerequisite for being considered a proficient language learner. However, unlike native language speakers, L2 learners seem to start by learning individual words and gradually build up larger language chunks, so it becomes particularly hard for L2 learners to establish strong associations among words to form collocations [50]. L2 learners tend to overuse combinations of words because of an influence of their L1, rather than store and produce native-like combinations of words as collocations [15,51]. Viewed from a formal pedagogical perspective, some of the problems L2 learners experience with L2 collocations may be caused by ineffective teaching. Some language teachers tend to focus on teaching individual words and often fail to raise learners’ awareness of collocations because they lack suitable teaching materials, such as written corpora, cloze tasks, and translation tasks [5]. A review of the literature on collocation learning strategies found that many previous studies [2,52,53] focused on the intentional learning of collocations through explicit instruction. A similar number have investigated collocation learning that occurs incidentally through reading [54,55,56] or listening [56]. However, we are not aware of any previous studies or language teaching guides that have introduced the KWM as a potential strategy for learning English collocations. The outcome of this current study may show the efficiency of the KWM and may benefit both language teachers and leaners in learning English collocations.

### 2.2. Theoretical Framework: Semantic Maps and Dual Coding Theory

When discussing or advocating particular learning strategies, a clear theoretical framework should be provided to help explain to the intended audience of the research what is being claimed and what is being discussed [57]. Although the KWM’s effect on individual word learning of both depth of vocabulary (the richness of vocabulary knowledge) and breadth of vocabulary knowledge (the number of vocabulary words known by a learner) has been examined in previous studies [58], the KWM has not been considered as a potential collocation learning strategy. A theoretical framework involving the semantic maps and dual coding theory was used to aid in explaining the possible reasons why the KWM is an effective strategy in facilitating English collocations learning. 

**Figure 1:**
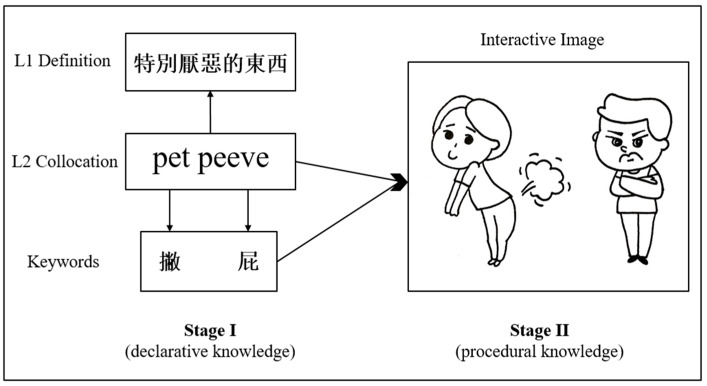
Semantic map of the application of the KWM to learn an English collocation.

Gage and Berliner [59] defined semantic maps as “organized visual and verbal maps of the declarative and procedural knowledge to be remembered” (p. 284). Declarative knowledge could be represented as facts, definitions, theories, and skills; procedural knowledge could be described as a type of knowledge that involves further learning elaboration and is “directly embodied in procedures” [60] (p. 369) for performing declarative knowledge [60,61]. When applying the KWM in learning new vocabulary, semantic associations are accessed and elaborated to facilitate learning the targets [62,63]. Therefore, it is easier for language learners to acquire vocabulary with tight connections in their semantic network. In Figure 1, we present an example of how the KWM can be used to learn the English collocation “pet peeve”. 

In this example, when learners apply the KWM to learn the collocation “pet peeve,” two stages are involved: stage one is recalling the L1 Chinese definition “tèbié yànwù de dōngxī” (meaning “something that a particular person finds especially annoying”) that corresponds to two keywords: “piē” (meaning “throw”) and “pì” (meaning “fart”); stage two is retrieving the interactive image linking the two keywords and the L2 English collocation: The man finds people who fart especially annoying.

In addition, dual coding theory explains how the human brain stores information [59]. Paivio [64] holds a view that the things we see are stored in visual form and the things we hear and read are stored in verbal form. Gage and Berliner [59] reviewed Bower, Karlin, and Dueck’s [65] study claiming that information is stored in visual/verbal form or both and also stored by its meaning. Dual coding theory helps to explain the effectiveness of the KWM. The KWM combines verbal word form, visual/pictorial, and meaning for the learning of vocabulary items, which suggests that this strategy would be more effective in comparison with the RLM that uses only verbal word form, only visual word form, or only the combined verbal and visual word forms to store and retrieve vocabulary items [59]. Furthermore, positive results of foreign vocabulary learning using the KWM have been uncovered by most previous studies that have aimed at investigating its effectiveness [39,40,62,66,67], which could be explained by the KWM’s advantage of combining verbal and visual forms with meaning. This advantage also explains why the KWM was used for deliberate vocabulary learning in the previous studies [62,67].

While the lion’s share of previous research [30,35,68] shows an indication that the KWM is superior to the RLM for long-term retention of vocabulary, it has yet to be investigated whether the same trend will be shown for the learning of English collocations. Lastly, while native language keywords in a number of different languages (e.g., Thai [69], Spanish [24], English [21], and Turkish [31]) have been used to learn various L2 vocabulary, no study was found where the foreign language learners spoke Mandarin Chinese as an L1 that used Mandarin Chinese keywords to facilitate the learning of L2 English collocations. Therefore, this study addressed these gaps in the literature by examining whether the KWM facilitates the learning of English collocations for this specific EFL learner population. The potential for the KWM to aid in the long-term retention of English collocations is also examined through the comparison to the RLM.

### 2.3. Role of KWM and RLM in Developing Vocabulary-Collocation Proficiency of EFL Learners

Since Atkinson’s [22] early study, researchers have compared the KWM’s effectiveness with many other VLSs [23,24,35,70]. The KWM has excelled over other picture-based strategies [26], semantic-based strategies [26,31], L1 context-embedding strategies [14,28,66,71], and self-selecting of VLSs [14]. Previous studies comparing the KWM and the RLM have found the KWM to not only be more effective for long-term retention [35] but also more effective “in accelerating learning speed and boosting immediate recall of second-language vocabulary” [68] (p. 1). Although positive learning results of the application of the KWM have been uncovered by previous studies [29,39,40,66,67], a few studies have found the RLM more effective than the KWM [72,73]. Still, other studies have found no significant difference in learning outcomes from the application of these two VLSs [74]. Accordingly, the current study eliciting both quantitative and qualitative data may assist in teasing apart the inconsistencies found among these previous results.

## 3. Methodology

### 3.1. Participants

Three groups of participants were recruited: pilot study group (*n* = 15), KWM group (*n* = 15), and the RLM group (*n* = 15). All of the participants were required to complete an online questionnaire to verify that they met the following participant requirements: (1) non-English major undergraduates from the same comprehensive university in Macau; (2) Mandarin-dominant speakers that have learned English as their L2 for at least ten years; and (3) English abilities were equivalent to B1 (an intermediate level of proficiency in English) [75] in the Common European Framework Reference (CEFR) [76]. The participants’ scores of four major recognized English tests were used as the reference to justify that the participants’ English levels were equal to the B1 level: TOFEL ITP (460), TOEFL iBT (42), TOEIC (275), and IELTS (4.0 to 5.0) [76].

The pilot study group was recruited for the purpose of ensuring that the participants in the formal experiment would not have familiarity with the target collocations in this study. All participants in the pilot study group did not take any part in the formal experiment but only took a receptive recognition test. The results of the pilot study group were used for finalizing the target collocations and revising the receptive recognition test items used for the formal experiment. The KWM group and the RLM group were recruited for the formal study.

### 3.2. Criteria for Selecting Target Collocations

The target collocations were selected based on three criteria: (1) Targets were restricted to incongruent collocations, because they are more difficult to learn than congruent collocations [7,77]. (2) Targets were further limited to verb–noun and adjective–noun collocations, as they are the most researched collocation types and they are ubiquitous, appearing in various spoken and written language contexts [5,7]. (3) Targets all appeared in one corpus-based collocations dictionary [78], one English–Chinese bilingual dictionary [79], and one more than 560-million-word corpus [80]. 

Twenty-four potential items were selected and included in a candidate pool, which were further verified for incongruency by three bilingual Mandarin–English professors of English education. Then, the pilot study group was asked to sit a receptive recognition test for the potential targets. After the researcher read aloud the description of the experiment to the participants in the pilot study group, twenty minutes were provided to finish the test. All participants completed the assessment independently. Only potential target collocations in which 10% or less of the pilot study group participants received a correct score were selected as a target collocation for the formal experiment. This process resulted in ten target collocations: five verb–noun collocations and five adjective–noun collocations (See Appendix A).

### 3.3. Measurement Instruments

Different types of vocabulary knowledge can be measured by different assessment instruments [40]. It has been shown that receptive recognition is beneficial to the acquisition of receptive knowledge, while productive recall facilitates the gains of productive knowledge [81]. Therefore, to measure both receptive and productive knowledge of the target collocations, one receptive recognition multiple-choice test and one productive recall translation test were constructed for this study. Participants were required to sit a receptive recognition multiple-choice test and productive recall translation test as a pre-test, immediate post-test, and delayed post-test (see Appendix B and Appendix C). 

For the receptive recognition test, the participants were required to choose one of four options in Chinese corresponding to the target English collocation. An example is shown below:

( ) 1. broad daylightA.一片光明B.阳光普照C.光天化日D.万丈光芒

All multiple-choice items were “marked dichotomously with 1 point for a correct answer and 0 points for an incorrect answer”. The minimum and maximum scores on this receptive recognition test were 0 points and 10 points, respectively.

For the productive recall test, the participants needed to translate the target items from Chinese into English in the following way: *光天化日 b________ daylight*. The first letter of the adjective or the verb was provided to avoid possible but undesirable answers [14]. In this example, learners were required to write “road” to complete the target collocation: *光天化日 broad daylight.* The items in the productive recall test were in a different order than the items on the receptive recognition test to avoid the risk that the latter test’s answers may be affected by the earlier test [40]. The productive recall test was marked with 1 point for a correct answer and 0 points for an incorrect or blank answer. Incorrectly spelled answers received 1 point if they contained an inflected form. The minimum and maximum scores on this productive recall test were 0 points and 10 points, respectively.

### 3.4. Post Hoc Interviews

Additional data elicited from one-on-one post hoc interviews with the RLM group (*n* = 15) and the KWM group (*n* = 15) were gathered after the delayed post-test to gain a deeper understanding of the learners’ perceptions of the RLM and the KWM. In order to compare the participants’ perceptions of the RLM and the KWM, both groups were required to participate in the interviews. All fifteen of the original fifteen RLM group (100%) participants and all fifteen of the original fifteen KWM group (100%) participants agreed to be interviewed. Each participant was interviewed individually, and all interviews were conducted in Mandarin Chinese (participants’ L1). Following the recommendations of Miles et al. [82], two cycles of coding were conducted to assist in the analysis of the qualitative interview data.

### 3.5. Procedures

**Figure 2:**
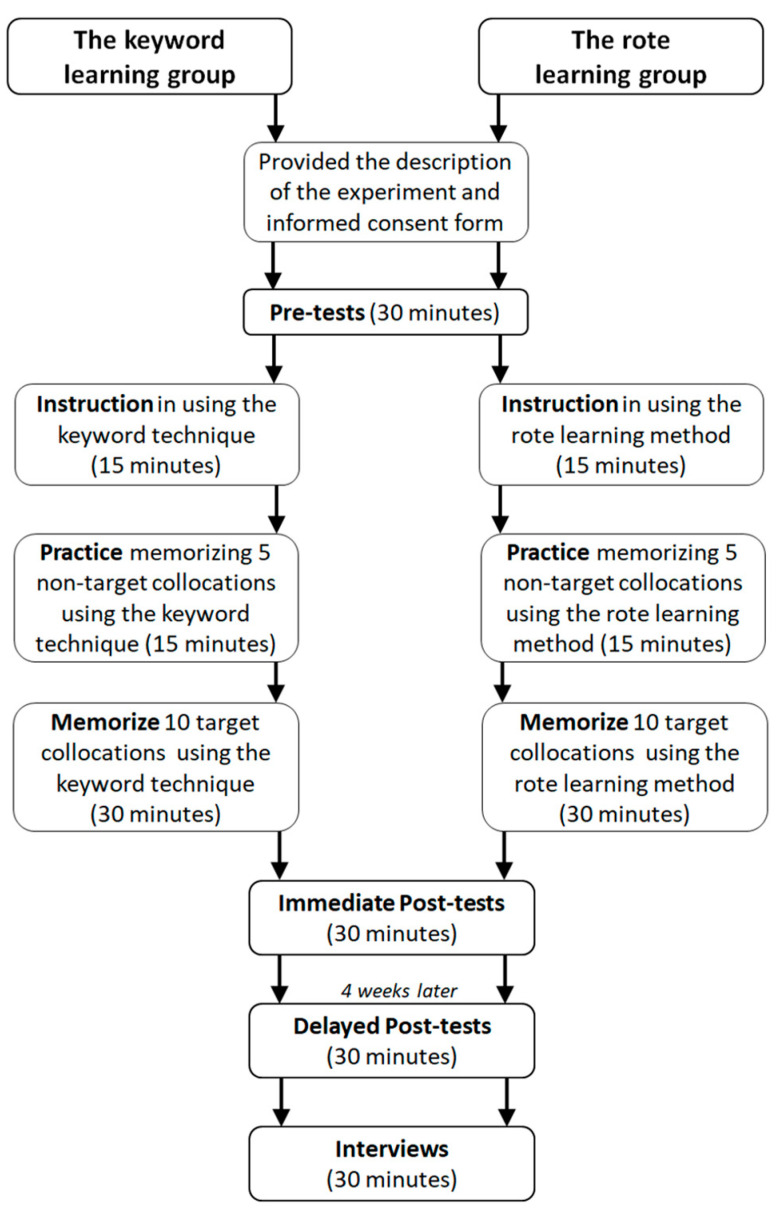
The procedures.

The procedures of the formal experiment are schematized in Figure 2 below. The pictures, L1 keywords, target collocations, and L1 definitions are provided in Appendix D.

Prior to starting the experiment, each participant was given the description of the experiment and the informed consent form. For the pre-test, participants were required to take one productive recall test and one receptive recognition test. If the receptive recognition test was conducted first, the participants may be cued with the target collocations. Therefore, the productive recall test was given before the receptive recognition test to avoid a learning effect of the target collocations from the completion of the productive recall test. After the pre-tests, the participants were given a detailed handout including instructions and directions regarding how to use the corresponding strategy. The instructions were read aloud by the researcher. After reading the instructions, the researcher further answered any questions posed by the participants. Then, the researcher provided participants with five nontarget collocations for practicing the respective strategy. The participants were asked to practice for fifteen minutes, wrote the five English collocations on a piece of paper, and then verbally stated their definitions. Furthermore, the participants elaborated and explained what they did to the researcher in the practice stage. After the practice, the participants were given ten target collocations to memorize. The participants sat an immediate post-test after the memorization phase. Then, four weeks later they sat a delayed post-test to assess the retention of the targeted collocations. During the immediate post-test and the delayed post-test, the participants completed the same two tests: one productive recall test and one receptive recognition test. After the delayed post-test, interviews were conducted independently with each participant to gather their perceptions of the respective strategy.

## 4. Data Analysis

### 4.1. Analysis of Quantitative Assessments Data

The participants’ assessment outcomes were analyzed using quantitative methods. Three variables were targeted in the analysis: group (the KWM group and the RLM group), time (pre-test, immediate post-test, and delayed post-test), and type (verb–noun collocations and adjective–noun collocations). To answer the first research question, two 3 × 2 RM ANOVAs were conducted to examine the potential interaction effects between group and time. To answer the second research question, two additional 3 × 2 RM ANOVAs were conducted to examine the potential interaction effects between type and time for only the KWM group’s assessment results.

### 4.2. Analysis of Qualitative Interview Data

Descriptive coding was used in the first cycle of coding based on the interview questions, which allowed for the development of three categories for indexing: learning background; implementation of the experiment; and perceptions of the VLS. In vivo coding was also used to emphasize interviewees’ representative opinions and comments [83]. Pattern coding was used in the second cycle of coding to develop three major themes (see Table 1) based on the results gathered from the first cycle of coding. Furthermore, focused coding was used to place the three major themes under two focused categories: perception of the KWM and perception of the RLM (see Table 2).

## 5. Results

### 5.1. RQ1: Which VLS Is More Effective for Learning English Collocations: KWM or RLM?

Descriptive statistics of the two assessments at three different times are shown in Table 3.

**Figure 3:**
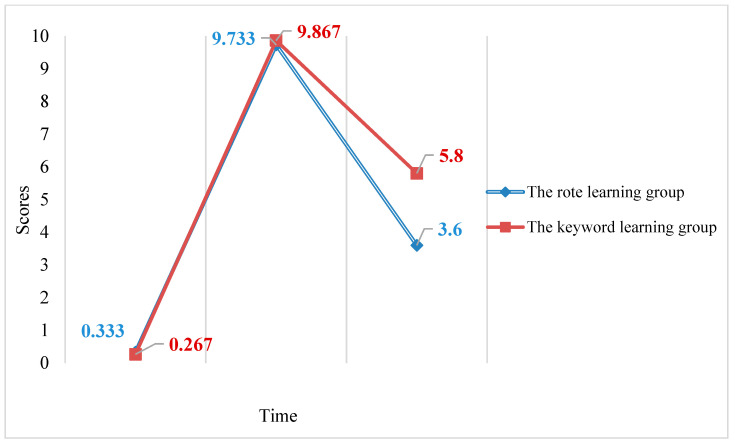
Mean pre-test, immediate post-test, and delayed post-test scores for the RLM group and KWM group’s productive recall test.

A 3 × 2 RM ANOVA examining the effects of time and group on the productive recall test scores found a statistically significant main effect for time (*F*2,27 = 4585.026, *p* < 0.05, partial eta squared = 0.997) indicating that time had a large effect on productive recall test scores. A statistically significant main effect for group (*F*1,28 = 13.785, *p* = 0.001, partial eta squared = 0.330) was also found, indicating that group had a large effect on productive recall test scores. A significant interaction effect between time and group (*F*2,27 = 13.835, *p* < 0.05, partial eta squared = 0.506) was found, indicating that time and group had a large interaction effect on productive recall test scores. The interaction accounts for 50.6% of the variance in the productive recall test scores (see Figure 3). Simple main effects were investigated to uncover the nature of the interaction. Bonferroni post hoc tests showed a statistically significant difference (*p* < 0.05) between the two groups’ delayed post-test scores. Two one-way RM ANOVAs were further conducted, finding a significant effect of time for the RLM group’s productive recall test scores (*F*2,13 = 2440.911, *p* < 0.05, partial eta squared = 0.997) and also for the KWM group’s productive recall test scores (*F*2,13 = 2496.589, *p* < 0.05, partial eta squared = 0.997). 

According to the productive recall results, immediate recall was better than delayed recall across both training approaches. Both the RLM and the KWM showed the same pattern in productive collocation knowledge: both groups’ productive knowledge increased after the use of the respective strategy and then both decreased over time. Furthermore, the KWM group showed a higher performance in the delayed post-test, which indicated that the KWM was more effective than the RLM in facilitating long-term retention of English collocation knowledge.

**Figure 4:**
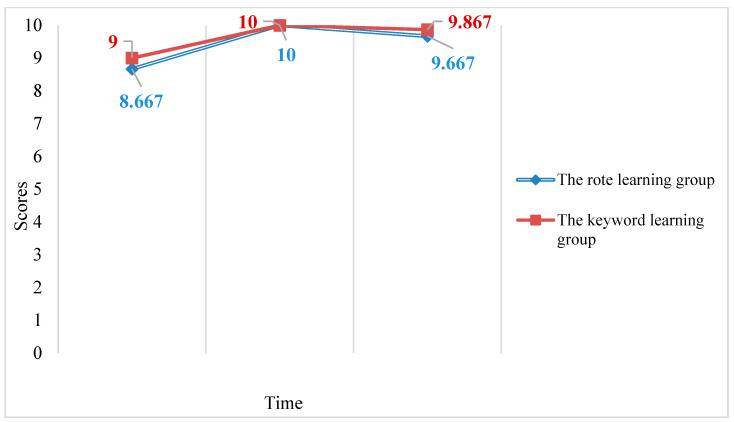
Mean pre-test, immediate post-test, and delayed post-test scores for the RLM and KWM groups’ receptive recognition test.

A 3 × 2 RM ANOVA examining the effects of time and group on the receptive recognition test scores only found a statistically significant main effect for time (*F*2,27 = 13.034, *p* < 0.05, partial eta squared = 0.491), indicating that time had a large effect on receptive recognition test scores (see Figure 4). Simple main effects were investigated to uncover the nature of the time variable’s effect. Bonferroni post hoc tests showed no statistically significant difference between the two groups’ pre-test, immediate post-test, or delayed post-test scores. Two one-way RM ANOVAs were further conducted finding a significant effect of time for the RLM group’s receptive recognition test scores (*F*2,13 = 6.101, *p* < 0.05, partial eta squared = 0.484) and also for the KWM group’s receptive recognition test scores (*F*2,13 = 7.964, *p* < 0.05, partial eta squared = 0.551) indicating that time had a large effect on receptive recognition test scores for both groups. 

According to the receptive recognition results, both the RLM and the KWM showed the same pattern in receptive collocation knowledge: both groups’ receptive knowledge increased after the use of the respective strategy and then both decreased over time. Furthermore, the KWM group and the RLM group showed a similar performance in the delayed post-test, which indicated the KWM was not more effective than the RLM for learning receptive collocation knowledge.

### 5.2. RQ2: Which Type of English Collocations Can Be Learned More Effectively by Using the KWM: Verb–Noun or Adjective–Noun Collocations?

Descriptive statistics of the two assessments at three different times for the KWM group organized by collocation type are shown in Table 4.

**Figure 5:**
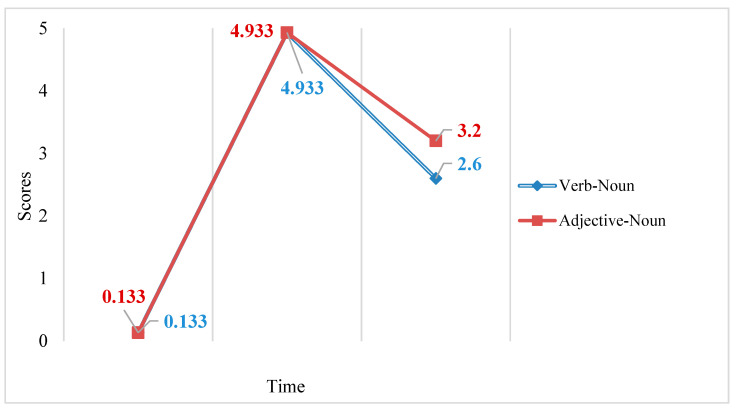
Mean scores of the KWM group’s productive recall pre-test, immediate post-test, and delayed post-test for verb–noun collocations and adjective–noun collocations.

A 3 × 2 RM ANOVA examining the effects of time and type on the productive recall test scores for the KWM group only found a statistically significant main effect for time (*F*2,27 = 1968.906, *p* < 0.05, partial eta squared = 0.993), indicating that time had a large effect on productive recall test scores (See Figure 5). Bonferroni post hoc tests showed no significant difference between verb–noun collocations and adjective–noun collocations’ pre-test, immediate post-test, or delayed post-test scores. While no significant difference (*t* = −1.775, *p* = 0.087, *df* = 27.511, 95% CI −1.293, 0.093) was found between the delayed post-test scores for verb–noun collocations (*M* = 2.600, *SD* = 0.986, *n* = 15) and adjective–noun collocations (*M* = 3.200, *SD* = 0.862, *n* = 15), there was a medium effect for type (*d* = 0.648). Two one-way RM ANOVAs were also conducted. A significant effect of time for verb–noun collocations’ productive recall test scores (*F*2,13 = 1072.783, *p* < 0.05, partial eta squared = 0.994) and for adjective–noun collocations’ productive recall test scores (*F*2,13 = 951.878, *p* < 0.05, partial eta squared = 0.993) were found, indicating that time had a large effect on productive recall test scores.

According to the productive recall results, both verb–noun collocations and adjective–noun collocations showed the same pattern in productive collocation knowledge. Productive knowledge of both types of collocations first increased after use of the KWM and then decreased over time. Furthermore, a medium effect size for type was found, which suggests adjective–noun collocations were retained better than verb–noun collocations on the delayed post-test.

**Figure 6:**
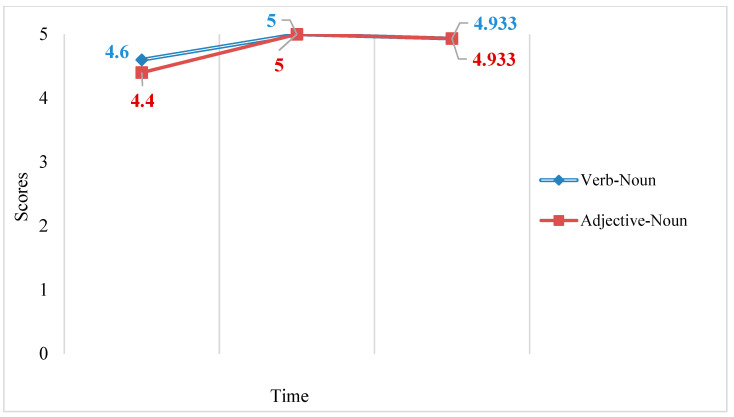
Mean scores of the KWM group’s receptive recognition pre-test, immediate post-test, and delayed post-test for verb–noun collocations and adjective–noun collocations.

A 3 × 2 RM ANOVA examining the effects of time and type on the receptive recognition test scores of the KWM group only found a statistically significant main effect for time (*F*2,27 = 8.635, *p* < 0.05, partial eta squared = 0.390), indicating that time had a large effect on receptive recognition test scores (See Figure 6). Simple main effects were investigated to uncover the nature of the time variable. Bonferroni post hoc tests showed no significant difference between verb–noun and adjective–noun collocation scores for the pre-test, immediate post-test, or delayed post-test. Two one-way RM ANOVAs were further conducted finding a significant effect of time only for adjective–noun collocations’ receptive recognition test scores (*F*2,13 = 6.050, *p* < 0.05, partial eta squared = 0.482), indicating that time had a large effect on receptive recognition test scores.

According to the receptive recognition results, both verb–noun collocations and adjective–noun collocations showed the same pattern in receptive collocation knowledge. Receptive knowledge of both types of collocations first increased after used of the KWM and then decreased over time. Furthermore, the lack of a statistically significant effect for type suggests that the KWM was not more effective for learning verb–noun collocations than adjective–noun collocations.

### 5.3. RQ3: How Did Learners Perceive the KWM and the RLM for Learning English Collocations?

A matrix is used to display the qualitative interview data (*n* = 30) focused on learners’ perceptions of two VLSs, namely, the KWM and the RLM (see Table 5). The learners’ perceptions were arranged under three themes (difficulties using the target VLS; advantages of the target VLS; and disadvantages of the target VLS) used for the discussion found in Section 5.3.

#### 5.3.1. Learners’ Perceptions of the KWM

The qualitative interview results showed that the participants were not aware of the KWM and had never applied it to learn English collocations in their previous English learning. Some participants (*n* = 9) in the KWM group had used other VLSs to learn English vocabulary, for example, using images (*n* = 5), using L1 homophonic words (*n* = 3), and using stories (*n* = 1) to associate L2 and L1 words. In addition, the participants reported two main difficulties in using the KWM.

1. Hard to recall some collocations on the delayed post-test (*n* = 8).
*K003: The difficulty was that some collocations were hard to be recalled when having the third test.*

2. Difficult to memorize the collocations based on provided pictures (*n* = 5).
*K004: I felt the pictures were hard to recall. I felt it clearer to memorize [the collocations] through memorizing the keywords. The pictures were easy to be forgot.*

Some participants held positive feelings towards the KWM and deemed it helpful (*n* = 13), intriguing (*n* = 8), and effective (*n* = 9). Most of the participants (*n* = 9) deemed the KWM an effective learning strategy. Moreover, unique characteristics of the KWM endowed other advantages:

1. The KWM is beneficial to learn abstract words (*n* = 2).
  *K015: It is a good strategy to learn some abstract…words.*

2. One keyword corresponds to one English word (*n* = 2).
*K015: I think this strategy is pretty effective for collocation learning. A similar strategy I have seen before is to split a word into syllables and then have a keyword for every syllable. I would feel that words are very scattered by using a strategy like that when I am learning. But for learning collocations, I’ve seen the examples you provided which are basically one keyword corresponds to one word, so that I think it is acceptable. There is an existing link between the two words. Then when you read the keyword, you are unintentionally combining two words into a collocation. I feel that it is more comfortable for me rather than [the way that] one word is split into several syllables.*

In contrast, some participants also held negative feelings towards the KWM and thought it troublesome (*n* = 5) and fantastical (*n* = 2). Two more disadvantages of the KWM were mentioned by some participants as follows:

1. The learning process is slow (*n* = 2).
  *K001: The disadvantage is that it may take a lot of time. It may take more time to design pictures and keywords and then to memorize [collocations].*

2. The KWM is not suitable for learning all kinds of collocations (*n* = 1).
*K013: I don’t think that every collocation can use this method. It still has some limitations. I think the chosen collocations are quite coincidental, and it [the KWM] is actually not very practical. [I] feel that there are not a lot of words that can use [the KWM to memorize].*

#### 5.3.2. Learners’ Perceptions of the RLM

According to the interview results, all participants (*n* = 15) in the RLM group mentioned that the RLM was the most commonly used strategy they employed from primary throughout high school. Some participants (*n* = 10) also shared that their previous teachers required them to use the RLM when they encountered new words. Some difficulties in using the RLM were mentioned by the participants:

1. Hard to recall some collocations on the delayed post-test (*n* = 9).
  *R011: The difficulty was that I cannot recall clearly when having the third test.*

2. Chinese definitions are difficult to associate with the English collocations (*n* = 4).
*R015: I can recall some English [collocations], but it is hard to associate them with their Chinese [definitions]. Some provided Chinese definitions cannot help me to recall the English [collocations].*

Still, some participants (*n* = 5) deemed this strategy helpful for learning English collocations. Specifically, some participants provided more detailed advantages as follows:

1. The RLM combines reading and writing to memorize (*n* = 2).
  *R002: Using the [rote learning] method can help you memorize faster. Because you read it out and write it out, which may bring about a deeper impression.*

2. The RLM is effective for memorizing collocations in a short period of time (*n* = 2).
*R008: I think this [rote learning] method is certainly very useful in a short period of time.*

In contrast, participants who thought the RLM was troublesome (*n* = 5), boring (*n* = 3), inflexible (*n* = 2), and torturous (*n* = 1) held more negative feelings than positive feelings about the RLM. For example:

*R012: The memorization process of this [rote learning] method is very torturous. Because if you want to totally remember, a large number of repetitions would be needed.*

## 6. Discussion

The findings reported in this study present new contributions to the VLS research field. At the time of writing, no other EFL study was found that investigated the effect of the KWM on the learning of English collocations. The present findings indicate that the KWM and the RLM do have effects on the learning of English collocations. The implication of these effects is discussed as follows.

### 6.1. Effect of the KWM and the RLM on Collocation Learning

According to the productive recall results, the KWM was more effective than the RLM in facilitating long-term retention of English collocation knowledge. The long-term learning effect resulting from the use of the KWM could be explained by Paivio’s [64] dual coding theory. The KWM facilitates learning through the utilization of the imagery system that integrates both verbal and nonverbal representations, while the RLM only promotes the use of verbal representations. Using both verbal and nonverbal codes, which evoke deep levels of knowledge processing [84,85], could produce better recall than only using a verbal code. Furthermore, the present study’s results are in accordance with previous studies, which found the KWM to have a significant effect on the acquisition of productive vocabulary knowledge to a greater extent than the RLM [35,68]. These previous studies were only concerned with vocabulary learning [14,22,23]; however, the results of this present study revealed that the KWM is also effective for English collocation retention. 

According to the receptive recognition results, the KWM was not more effective than the RLM for learning English collocations. The present study is in accordance with previous research findings that found the use of the KWM did not affect learning significantly more than with the use of the RLM [73]. In addition to the use of a productive recall assessment, the present study also used a receptive recognition assessment to investigate long-term retention; however, a large number of previous studies used only a receptive recognition assessment to measure vocabulary growth [28,35,38,67]. Previous studies may not have always been able to show conclusive results, as they only used one type of assessment, so the present study has provided more robust findings as data were collected using two different assessments that tapped into two different types of vocabulary knowledge. Although there was no significant difference found between the two groups’ receptive recognition scores during the three testing times, a significant increase (*p* < 0.017) in collocation knowledge between the pre-test and immediate/delayed post-test scores was shown for both groups, indicating and increase in collocation knowledge after the application of both strategies.

Both the KWM group and the RLM group had the same pattern showing productive and receptive knowledge increased after the use of the respective strategy and then both decreased over time. The forgetting happened for both the KWM group and the RLM group, because the participants encountered the target collocations for only a single learning session. This similar phenomenon was also found in previous studies [14,67,73], where an increase in participants’ vocabulary knowledge was shown after the use of VLSs and then decreased at the time of the delayed post-test. The participants in the current and previous studies may have been unable to retrieve the target collocations because of a failure in their information search [59]. However, a KWM-RLM difference was found in the delayed post-test but not in the immediate post-test, which indicated less forgetting occurred for the KWM group in terms of learning English collocation over a long period of time. Less forgetting occurred for the KWM group because the participants in the current study were able to retrieve different types of information that they had linked together, including verbal representations and visual/pictorial representations.

Furthermore, a statistically significant difference between the KWM and the RLM groups’ delayed post-test productive recall assessment scores was found, while no significant difference was found for the two groups’ delayed post post-test receptive recognition assessment scores. One possible reason for this difference could be that the two assessments investigated two different types of collocation knowledge. When a participant answers a productive recall item, he or she needs to produce the target item based on the meaning provided, while a participant only needs to choose the meaning of the target item when answering a receptive recognition test item. Therefore, context may have been provided in the receptive recognition test, leading participants in this study to feel the receptive recognition test was easier to complete. This result could have also been due to a practice effect caused by the order of the tests. Because participants took the productive recall assessment first and then took the receptive recognition assessment, they might have been cued in the first assessment to be able to choose the correct answers on the second assessment.

### 6.2. Effect of the KWM on Learning Different Types of Collocations

According to the productive recall results, a medium effect size for type was found between the learning of the verb–noun or adjective–noun collocations by using the KWM, which suggests adjective–noun collocations were retained better than verb–noun collocations. Similar to the current research finding, Paivio and Desrochers [86] found that a picture version of a mental image shows an advantage in learning nouns and adjectives, while a sentence version of a mental image works better for verbs and adverbs. Because a picture version of a mental image facilitated the learning of concrete words that are easier to be imagined, such as nouns and adjectives, a picture version of a mental image was used in this study, which could be seen as the explanation of the result that adjective–noun collocations were retained better than verb–noun collocations on the delayed post-test.

According to the receptive recognition results, no significant difference was shown between the learning of English verb–noun or adjective–noun collocations using the KWM. The present study results indicated that use of the KWM had a similar effect on the learning of verb–noun and adjective–noun collocations. Receptive knowledge of both types of collocations first increased after participants applied the KWM and then decreased over time. Therefore, the results indicate that the use of the KWM has a similar effect for the learning of verb–noun and adjective–noun collocations. Previous studies that investigated the learning effects of the KWM focused on different types of L2 vocabulary or L1 keywords, for example, abstract words vs. concrete words [39], high imageability words vs. low imageability words [14], and verb keywords vs. noun keywords [87]; however, no previous study has attempted to investigate the use of the KWM for the learning of different types of collocations. Those previous studies focused on the learning of collocations without the application of the KWM only targeted certain types of collocations as targets, for example, verb–noun collocations [2,7,11,48], adjective–noun collocations [7,47,48], noun–verb collocations [88], and verb–adverb collocations [88]. However, unlike the present study, none of these previous studies attempted to apply the KWM as a possible facilitator in the learning of English collocations.

### 6.3. Learners’ Perceptions of the KWM

The qualitative interview results show that participants had never applied it to learn English collocations in their previous English learning. The previous literature [25,39,67] has criticized the KWM as not being a familiar VLS, because it is seldom introduced in foreign language teaching. Moir and Nation [89] found that learners were unwilling to apply the KWM even though they had been instructed and taught by language teachers to do so. Ellis and Beaton [87] stated that the less the overlap between the familiarity of features between L1 and L2, the harder it will be for language learners to learn words using the KWM. 

The participants reported two main difficulties in using the KWM. The first one is that many participants (*n* = 8) were hard to recall some collocations on the delayed post-test. This difficulty was the most frequent difficulty mentioned by the participants in both the KWM group and the RLM group, which can also be verified by the quantitative assessment results that show a decrease in the delayed post-test scores. The second difficulty is that some participants (*n* = 5) found it difficult to memorize the collocations based on the provided pictures. They mentioned that with the provided pictures they found it difficult to imagine a relationship between the keywords and collocations. Atkinson [22] pointed out that a sentence version of the mental image may be more appropriate than a picture version, because with provided pictures learners may find it difficult to associate the keyword with the target item. However, Pressley et al. [90] found no statistical difference between a sentence version keyword group and a picture version keyword group. Clark and Paivio [84] explained that imagery processing may be affected by “variation among people in the tendency and capacity to use imagery” (p. 156). This could explain why some participants in the current study used imagery easily to memorize the target collocations, while others felt imagery was difficult.

In terms of the advantages of the KWM, some participants (*n* = 2) perceived that the KWM is beneficial for learning abstract words. From the participants’ perceptive, the KWM made it easier to learn abstract words in comparison with using other learning strategies [25]. According to the psycholinguistic literature, researchers have claimed that concrete words can be better memorized than abstract words [91], and KWM can help learners to come up with images to make abstract words become more concrete. Although the KWM enjoys a good reputation, language teachers and learners have seldom applied this strategy when they learn or teach vocabulary [39,40] and preferred to apply and teach simpler strategies like the RLM [32,92]. The participants provided reasons to explain this phenomenon. Most of the participants mentioned that they probably would not have been willing to apply the KWM in learning English vocabulary, as they would have thought one keyword would have been needed for each English syllable in a target word. However, after they were instructed on how to use the KWM in learning English collocations, they not only deemed the KWM effective for learning vocabulary but thought it might be even more effective for learning collocations. They explained that having one L2 keyword to correspond to one English word really did help them to memorize target collocations (*n* = 2). When they learned a word like “ambulance”, for example, they would associate the four Chinese keywords “ǎn bùnéng sǐ” (meaning “I cannot die”) with each of the four English syllables in the target word “ambulance”. Then, the learners employed the four Chinese keywords to evoke an image: “A man said, ‘I cannot die.’ in an ambulance”. Sometimes keywords did not correspond to all of the syllables in a target vocabulary item. However, when learning English collocations, for example, “foot bill”, the participants only needed to associate one Chinese keyword “fù” (meaning “pay”) with one English word “foot” and associate another Chinese keyword “bì” (meaning “currency”) with the English word “bill” and then employ the two keywords with a picture provided to evoke an image: “A man pays the bill with his foot”. 

In contrast, some participants also held negative feelings towards the KWM and thought the learning process is troublesome (*n* = 5), fantastical (*n* = 2), and slow (*n* = 2). For some language learners, learning a new word using the KWM may be seen as “an incremental process” [39] (p. 212). When using the KWM, learners not only need to memorize an L2 word and its L1 definition but also need to remember a keyword and a mental image. The KWM may require learners to spend more time in the beginning of learning, but just as the quantitative data show, more time invested may result in stronger retention. Additionally, one participant felt that the KWM is not suitable for learning all kinds of collocations, which may because the effectiveness of the KWM largely depends “on the degree of concreteness and imageability of the target words” [93] (p. 233). Ellis [94] found that the beneficial effects of the KWM decreased with target vocabulary “involving low levels of concreteness and imageability” [93] (p. 230). Therefore, although the KWM may not be suitable for learning all collocation types, it is still beneficial for learners to know that it may be more effective for learning collocations that contain words involving high levels of concreteness and imageability.

### 6.4. Learners’ Perceptions of the RLM

Some difficulties in using the RLM were mentioned by the participants. Similarly, the most frequent difficulty mentioned by the participants (*n* = 9) was that they were hard to recall some collocations on the delayed post-test, which can be verified by the quantitative assessment results that showed a decrease in the delayed post-test scores. Furthermore, when using the RLM, some participants (*n* = 4) deemed that Chinese definitions are difficult to associate with the English collocations. Unlike the KWM group, participants in the RLM group could not trace keywords or pictures from their memory to help them to recall the target collocations but instead had to recall the English collocations based only on the given Chinese definitions. Thus, four weeks after the learning session, most participants in the RLM group had a hard time recalling the target collocations.

However, some participants (*n* = 2) still felt that the RLM is an effective strategy to use when time is limited, which is also much easier to apply, compared with other complex strategies they have used before. When using the RLM, they only needed to combine reading and writing to memorize the collocations. Some other participants (*n* = 2) mentioned that the RLM is effective for short-term learning but may be easily forgotten, which supports the quantitative data collected through the two assessments.

Participants who thought the RLM was troublesome (*n* = 5), boring (*n* = 3), inflexible (*n* = 2), and torturous (*n* = 1) held more negative feelings than positive feelings about the RLM. Gu and Johnson [36] found learners more commonly preferred to use rehearsal strategies like the RLM than other strategies. Unfortunately, rehearsal strategies are less efficient than encoding strategies such as the KWM at helping learners retain vocabulary knowledge over time. In addition, strategies such as the RLM may be considered less enjoyable by learners. In the participants’ views, the RLM was seen as a decontextualized learning strategy. Previous studies argued that the RLM impedes creative learning [36]. 

## 7. Limitations and Future Studies

The findings of this study could be used for future investigations of collocation learning through the use of the KWM. The limitations of this study are presented below. Firstly, the order that the two assessments were administered to the participants may have influenced outcomes shown on the second assessment. Because the L1 Chinese definitions were provided in the productive recall test, the participants might have been cued to choose correct answers on the receptive recognition test. This assessment order might have also led to a ceiling effect for the receptive recognition test. A future study could be designed providing multiple-choice options in the L2 as a way to possibly avoid this limitation. In this way, the participants would not be exposed to the L1 definitions of targets prior to completion of a receptive recognition test. Secondly, familiarity towards the VLS may have affected on collocation learning outcomes. According to the qualitative results, participants in the keyword learning group had not applied the KWM in learning collocations; however, participants in the RLM group deemed the RLM as the most commonly used strategy. Therefore, the unfamiliarity towards the KWM may have led to the quantitative result of no significant difference being found between the two VLSs on the immediate post-test. Thirdly, while the target collocations were limited to incongruent collocations and the phraseological categories of verb–noun and adjective–noun, the length of the words, frequency of the collocates, and “the degree of concreteness and imageability of the target” [93] (p. 233) collocations were not taken into consideration. A future study could be designed considering a more balanced selection of a larger target item dataset for target items’ length, frequency, concreteness, and imageability. Fourthly, one other limitation deals with the scope and the target participants of this study. Participants’ individual differences may have affected imagery processing when using the KWM. According to Clark and Paivio [84], imagery processing may be affected by “variation among people in the tendency and capacity to use imagery” (p. 156). A future study could be designed taking the learners’ individual differences into consideration.

## 8. Implications and Conclusions

The development of EFL students’ collocation learning is very uneven [54]. The results of the productive recall test suggest that the use of the KWM to learn collocations can bring about long-term retention that is more robust than compared to that of the RLM. Although the differences between Chinese and English may cause difficulties in the design of high-quality keywords [14], L1 Chinese language learners still need to be encouraged to use the KWM to learn collocations. Furthermore, when leaners need to come up with appropriate keywords, they may have deeper impressions of the L2 target items due to the elaborative learning process [16,25]. According to the qualitative interview results, learners held more positive feelings towards using the KWM than the RLM. The positive beliefs about the KWM suggested that the KWM may help learners to consolidate knowledge and deepen their understanding of English collocations. 

Although the KWM has been recommend by many researchers, it is still rarely advocated by foreign language instructors [25,39]. Therefore, it is important that teachers are aware of the long-term learning effect of the KWM. Teachers are suggested to discuss and evaluate language learning strategies used by their students using the following steps: (1) Teachers are recommended to show examples of the KWM and offer it as one of the options to learn L2 collocations. (2) Teachers can provide target collocations and then encourage students to come up with their own keywords and mental images. (3) Teachers can further ask students to compare and discuss the keywords and mental images with their classmates. During the discussion phase, learners would not only be able to learn the target collocations receptively but also practice them productively. (4) Teachers are recommended to evaluate the keywords and the mental images created by the students. Teachers need to provide useful advice as well as to remind students to be aware of both the values and the limitations of the KWM.

## Figures and Tables

**Table 1:** Second cycle of coding: major themes.

Major Themes	Abbreviation
Theme 1: Difficulties using the target vocabulary learning strategy	VLS-D
Theme 2: Advantages of the target vocabulary learning strategy	VLS-A
Theme 3: Disadvantages of the target vocabulary learning strategy	VLS-DA

**Table 2:** Second cycle of coding: focused categories.

Focused Categories	Abbreviation
Perceptions of the keyword method	PVLS-KWM
Perceptions of the rote learning method	PVLS-RLM

**Table 3:** Descriptive statistics for the RLM and KWM groups’ productive recall and receptive recognition tests.

	Tests	RLM Group (*n* = 15)			KWM Group (*n* = 15)		
		M	SD	Scoring Ranges	M	SD	Scoring Ranges
Pre-test	Productive	0.333	0.488	0–1	0.267	0.458	0–1
	Receptive	8.667	1.543	5–10	9.000	1.000	7–10
Immediate post-test	Productive	9.733	0.594	8–10	9.867	0.352	9–10
	Receptive	10.000	0.000	10–10	10.000	0.000	10–10
Delayed post-test	Productive	3.600	1.502	1–6	5.800	1.146	4–8
	Receptive	9.667	0.488	9–10	9.867	0.352	9–10

Total possible score = 10.

**Table 4:** Descriptive statistics for the KWM group’s productive recall and receptive recognition tests with a focus on two types of collocations.

	Tests	Productive Recall Test (*n* = 15)			Receptive Recognition Test (*n* = 15)		
		M	SD	Scoring Ranges	M	SD	Scoring Ranges
Pre-test	Verb–Noun	0.133	0.352	0–1	4.600	0.632	3–5
	Adjective–Noun	0.133	0.352	0–1	4.400	0.737	3–5
Immediate post-test	Verb–Noun	4.933	0.258	4–5	5.000	0.000	5–5
	Adjective–Noun	4.933	0.258	4–5	5.000	0.000	5–5
Delayed post-test	Verb–Noun	2.600	0.986	1–4	4.933	0.258	4–5
	Adjective–Noun	3.200	0.862	2–5	4.933	0.258	4–5

Total possible score = 5.

**Table 5:** Results of the qualitative interviews data.

Themes	Perception of the KWM	Perception of the RLM
Difficulties (VLS-D)	1. Hard to recall some collocations on the delayed post-test (*n* = 8); 2. Difficult to memorize the collocations based on provided pictures (*n* = 5).	1. Hard to recall some collocations on the delayed post-test (*n* = 9); 2. Chinese definitions are difficult to associate with the English collocations (*n* = 4).
Advantages (VLS-A)	1. Helpful (*n* = 13); 2. Intriguing (*n* = 8); 3. Effective (*n* = 9); 4. Is beneficial to learn abstract words (*n* = 2); 5. One keyword corresponds to one English word (*n* = 2).	1. Helpful (*n* = 5); 2. Combines reading and writing to memorize (*n* = 2); 3. Is effective for memorizing collocations in a short period of time (*n* = 2).
Disadvantages (VLS-DA)	1. Troublesome (*n* = 5); 2. Fantastical (*n* = 2); 3. Learning process is slow (*n* = 2); 4. Not suitable for learning all kinds of collocations (*n* = 1).	1. Troublesome (*n* = 5); 2. Boring (*n* = 3); 3. Inflexible (*n* = 2); 4. Torturous (*n* = 1).

## Data Availability

The data presented in this study are available upon request from the corresponding author. The data are not publicly available because of the restrictions set by the ethics committee review.

## Appendix A

**The Target Collocations List**

**No.**	**Category**	**Collocations**
1	Verb + Noun	foot bill
2	Verb + Noun	pick brain
3	Verb + Noun	pull face
4	Verb + Noun	seal fate
5	Verb + Noun	slip mind
6	Adjective + Noun	dead wood
7	Adjective + Noun	fat chance
8	Adjective + Noun	hidden agenda
9	Adjective + Noun	red tape
10	Adjective + Noun	top drawer

## Appendix B

**Receptive Recognition Multiple-choice Test for the Formal Experiment**
Multiple-choice Test
*Please write the letter in the space provided for the Chinese option that corresponds to the English collocation.*
请在下列每题的四个选项中选出与所给英语词组相对应的一个中文选项。
( ) 1. hidden agenda
A.隐藏的秘密 (hidden secret)
B.隐秘的动机 (a secret reason for doing something)
C.难言之隐 (secrets or problems one doesn’t want to reveal)
D.暗中的策划 (secret plan)
( ) 2. foot bill
A.欠账 (arrears)
B.要帐 (ask for a bill)
C.付账 (pay for something)
D.记账 (bookkeeping)
( ) 3. red tape
A.细枝末节 (minor details)
B.严重警告 (serious warning)
C.繁文缛节 (official rules and processes that seem unnecessary and delay results)
D.强制规定 (mandatory requirement)
( ) 4. pick brain
A.对某事挑剔 (picky about something)
B.征集意见 (solicit opinions)
C.冥思苦想 (think hard)
D.向某人求教 (to ask someone who knows a lot about a subject for information or their opinion)
( ) 5. fat chance
A.非常有可能 (very possible)
B.许多的机会 (many opportunities)
C.失去的机会 (lost opportunity)
D.没什么可能 (very little or no possibility)
( ) 6. top drawer
A.最优秀的 (the highest quality)
B.难以达到的 (hard to reach)
C.量身定制的 (tailor-made)
D.令人羡慕的 (enviable)
( ) 7. slip mind
A.忘记 (forget)
B.分歧 (disagreement)
C.走神 (distracted)
D.退步 (regress)
( ) 8. dead wood
A.不再有用的人或事物 (someone or something that is unwanted and unneeded)
B.无聊的人或事物 (someone or something that is bored)
C.过期的物品 (expired items)
D.死气沉沉的环境 (inanimate environment)
( ) 9. pull face
A.按摩脸 (massage face)
B.亮相 (appearance)
C.给脸色 (give someone attitude)
D.扮鬼脸 (to show a feeling such as dislike or disgust by twisting your face into an ugly expression)
( ) 10. seal fate
A.掌握命运 (take control of your destiny)
B.难以改变 (hard to change)
C.在劫难逃 (nothing can stop some unpleasant thing happening to you)
D.一帆风顺 (plain siling)
Note. English translation for readers’ references only. The version with Chinese options was provided to the research participants.

## Appendix C

**Productive Recall Translation Test for the Formal Experiment**
Chinese-to-English Translation Test
*Please translate the collocations from Chinese into English by filling in the blanks with the missing letters.*
请填上所缺字母，将所给中文翻译成英文。

1. 不再有用的人或事物 d__________ wood
  (someone or something that is unwanted and unneeded)
2. 忘记 s__________ mind
  (forget)
3. 在劫难逃 s__________ fate
  (nothing can stop some unpleasant thing happening to you)
4. 向某人求教 p__________ brain
  (to ask someone who knows a lot about a subject for information or their opinion)
5. 扮鬼脸 p__________ face
  (to show a feeling such as dislike or disgust by twisting your face into an ugly expression)
6. 繁文缛节 r__________ tape
  (official rules and processes that seem unnecessary and delay results)
7. 没什么可能 f__________ chance
  (very little or no possibility)
8. 最优秀的 t__________ drawer
  (the highest quality)
9. 付账 f__________ bill
  (pay for something)
10. 隐秘的动机 h__________ agenda
  (a secret reason for doing something)

Note. English translation for readers’ references only. The version with Chinese options was provided to the research participants.

## Appendix D

**Pictures of Ten Target Collocations**

1. foot bill

2. pick brain

3. pull face

4. seal fate

5. slip mind

6. dead wood

7. fat chance

8. hidden agenda

9. red tape

10. top drawer
